# Systematic and searchable classification of cytochrome P450 proteins encoded by fungal and oomycete genomes

**DOI:** 10.1186/1471-2164-13-525

**Published:** 2012-10-04

**Authors:** Venkatesh Moktali, Jongsun Park, Natalie D Fedorova-Abrams, Bongsoo Park, Jaeyoung Choi, Yong-Hwan Lee, Seogchan Kang

**Affiliations:** 1 Integrative Biosciences program in Bioinformatics & Genomics, The Pennsylvania State University, University Park, PA, USA; 2 Fungal Bioinformatics Laboratory, Seoul National University, Seoul, Korea; 3 Advanced Biomedical Computing Center, SAIC-Frederick, Frederick National Laboratory for Cancer Research, Frederick, MD, USA; 4 Department of Agricultural Biotechnology and Center for Fungal Pathogenesis, Seoul National University, Seoul, Korea; 5 Department of Plant Pathology & Environmental Microbiology, The Pennsylvania State University, University Park, PA, USA

**Keywords:** Cytochrome P450, Genome annotation, Clustering, Fungi, Evolution, Phylogenetics, Mycotoxin

## Abstract

**Background:**

Cytochrome P450 proteins (CYPs) play diverse and pivotal roles in fungal metabolism and adaptation to specific ecological niches. Fungal genomes encode extremely variable “CYPomes” ranging from one to more than 300 CYPs. Despite the rapid growth of sequenced fungal and oomycete genomes and the resulting influx of predicted CYPs, the vast majority of CYPs remain functionally uncharacterized. To facilitate the curation and functional and evolutionary studies of CYPs, we previously developed Fungal Cytochrome P450 Database (FCPD), which included CYPs from 70 fungal and oomycete species. Here we present a new version of FCPD (1.2) with more data and an improved classification scheme.

**Results:**

The new database contains 22,940 CYPs from 213 species divided into 2,579 clusters and 115 clans. By optimizing the clustering pipeline, we were able to uncover 36 novel clans and to assign 153 orphan CYP families to specific clans. To augment their functional annotation, CYP clusters were mapped to David Nelson’s P450 databases, which archive a total of 12,500 manually curated CYPs. Additionally, over 150 clusters were functionally classified based on sequence similarity to experimentally characterized CYPs. Comparative analysis of fungal and oomycete CYPomes revealed cases of both extreme expansion and contraction. The most dramatic expansions in fungi were observed in clans CYP58 and CYP68 (Pezizomycotina), clans CYP5150 and CYP63 (Agaricomycotina), and family CYP509 (Mucoromycotina). Although much of the extraordinary diversity of the pan-fungal CYPome can be attributed to gene duplication and adaptive divergence, our analysis also suggests a few potential horizontal gene transfer events. Updated families and clans can be accessed through the new version of the FCPD database.

**Conclusions:**

FCPD version 1.2 provides a systematic and searchable catalogue of 9,550 fungal CYP sequences (292 families) encoded by 108 fungal species and 147 CYP sequences (9 families) encoded by five oomycete species. In comparison to the first version, it offers a more comprehensive clan classification, is fully compatible with Nelson’s P450 databases, and has expanded functional categorization. These features will facilitate functional annotation and classification of CYPs encoded by newly sequenced fungal and oomycete genomes. Additionally, the classification system will aid in studying the roles of CYPs in the evolution of fungal adaptation to specific ecological niches.

## Background

Cytochrome P450 proteins (CYPs) are found in all domains of life [1] and represent one of the largest protein families. Their existence predates the emergence of oxygen-metabolizing life forms [2]. CYPs are defined by the absorption of light at 450nm by the heme cofactor, and oxidize a very diverse array of metabolic intermediates and environmental compounds. CYPs participate in a large number of primary, secondary and xenobiotic metabolic reactions [3].

The evolution of CYPs has been intimately intertwined with organismal adaptation to new ecological niches due to the roles of CYPs in the production of metabolites critical for specific processes such as pathogenesis, the utilization of specific substrates, and/or the detoxification of xenobiotics. Based on their roles in synthesizing or neutralizing toxic metabolites, many CYPs are hypothesized to have evolved through the chemical warfare waged among plants, animals, insects, and microbes [2,4]. In fungi, several CYPs have been implicated in pathogen virulence because they neutralize antifungal compounds produced by hosts [5-7]. Expansions and diversifications of several CYP families have been associated with the evolution of fungal pathogenicity [8]. Accordingly, functional and evolutionary analyses of CYPs have been useful in understanding the ecological specialization and functional diversification of individual fungal taxa [9].

The extraordinary functional and evolutionary diversity of fungal CYPomes presents a major hurdle to CYP classification [10]. Fungal CYPs share little sequence similarity, except for a few conserved residues that are characteristic of CYPs. The most conserved region is the binding domain for a heme cofactor. Substrate binding regions are much more variable but may possess a signature motif. This motif is often found in conjunction with one or more binding domains such as those for cytochrome b5, ferredoxin, and binding sites for the NADPH cytochrome P450 reductase that contains FAD (flavin adenine dinucleotide) and FMN (flavin mononucleotide) [11].

Another challenge in developing a comprehensive CYP classification system is the rapidly increasing number of sequenced fungal genomes. Currently, more than 250 genomes are present in the public domain [12,13], but this number is predicted to increase rapidly (e.g., http://1000.fungalgenomes.org). The rapid influx of genome sequences calls for robust computational tools that can effectively support large-scale comparative analyses of genomes and specific gene families.

The first nomenclature/grouping schema for CYPs, proposed by Nebert et al. in 1987 [14], was based on amino acid sequence similarity. According to this schema, any two CYPs with sequence identity greater than 40% belong to a single CYP family; and any two CYPs with sequence identity greater than 55% belong to a subfamily. Manually curated databases of CYPs in multiple kingdoms based on this approach (thereafter referred to as Nelson’s P450 databases) have been maintained at http://drnelson.uthsc.edu/CytochromeP450.html[15,16]. These databases also serve as a central repository of CYP nomenclature. Unfortunately, this schema cannot be efficiently used to curate and classify rapidly increasing CYPs uncovered through genome sequencing.

The clan system approach was developed to support higher-level grouping of families identified via the sequence similarity-based schema. This approach places all CYP families with a monophyletic origin into a single clan and has been successfully applied to classify CYP families in Metazoa [17] and four fungal species [10]. For example, if new CYPs had equal identity to two or more CYP families, they can be tentatively assigned to a clan in which these families belong. Since the introduction of the “clan concept” in 1998 to classify metazoan CYPs [17], additional clans in vertebrates (9), plants (11) [18], arthropods [19], bivalves (4), and fungi (115) [10] have been identified. However, the clan classification system has become problematic for classifying the pan-fungal CYPome, because the number of fungal CYPs is too large to conduct phylogenetic analyses efficiently. Automated clustering based on sequence similarity remains the gold standard for the rapid classification of large protein sets [20,21]. This approach does not require any prior knowledge and allows for rapid clustering of large protein families such as CYPs.

In 2008, we employed an automated clustering approach to build the Fungal Cytochrome P450 Database (FCPD) [22]. Since then the number of sequenced fungal genomes has increased substantially, which necessitated the improvement of our classification system. Additionally, the original FCPD classification generated several mega clusters, underscoring the need for optimizing clustering parameters.

Here we present FCPD release 1.2 ( http://p450.riceblast.snu.ac.kr) with an improved CYP classification pipeline based on the modified TRIBE-MCL algorithm. The pipeline allowed for a larger number of CYP families to be merged into existing clans as well as supporting the discovery of potential new clans. To aid functional annotation, putative functional roles were assigned to over 150 clusters based on their similarity to functionally characterized fungal CYPs. The families and clans are accessible through FCPD, which offers global viewing and analysis of fungal CYPs.

## Results and discussion

### Identification of CYPs and optimization of clustering parameters

**Figure 1:**
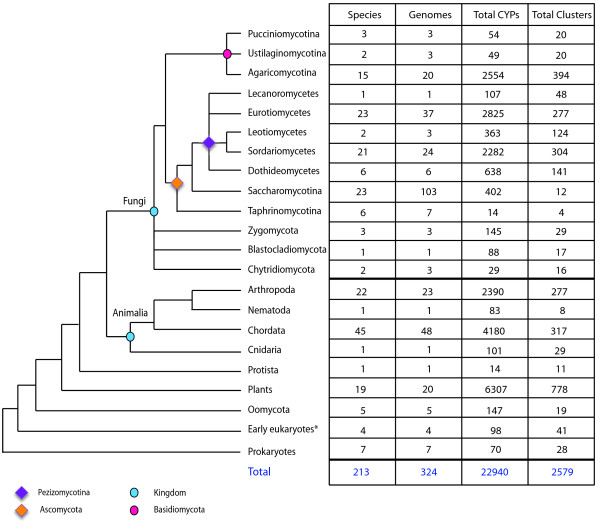
**Phylogenetic relationships among taxa in FCPD 1.2 and the number of CYPs and clusters in each taxon.** The tree topology based on Patterson and Sogin [23] is accompanied by a summary of the data archived in FCPD1.2. Each cluster may have CYPs from more than one phylum/subphylum. The number of clusters roughly corresponds to the number of CYP clans/families, thus illustrating the diversity of CYPs in each taxon.

We first extracted all proteins that contained Interpro ( http://www.ebi.ac.uk/interpro/) terms associated with CYPs from 324 genomes corresponding to 113 fungal and oomycete species, 94 other eukaryotic species, and six bacterial species (Figure 1) as previously described [22]. While our main focus has been on curating fungal and oomycete CYPs, CYPs encoded by other eukaryotic species and selected bacterial species were included to aid in comparative evolutionary studies across kingdoms. Although oomycetes are fungus-like in that they produce hyphae and spores, they reside in a more basally derived eukaryotic lineage that includes chromophyte algae (Figure 1). However, because mycologists have traditionally studied oomycetes, we analyzed CYPs from both true fungi and oomycetes. This data extraction resulted in 22,940 CYPs including 9,697 CYPs from fungi and oomycetes and 13,243 CYPs from other organisms (Figure 1). 

Extracted protein sequences were clustered using an optimized protocol based on reciprocal pair-wise BLASTp all-against-all comparisons [24] followed by Tribe-MCL clustering [21] (see Methods and Additional file 1 for details). The revision of the original clustering pipeline used to build FCPD [22] was motivated by a few factors, including the presence of many mega clusters with over 100 members, singlet clusters, and clusters that did not match families in Nelson’s P450 databases. While there are no absolute “best” criteria to optimize clustering, our main goal was to achieve more uniform grouping by minimizing the fractions of very large (>100 members) and singlet clusters.

**Figure 2:**
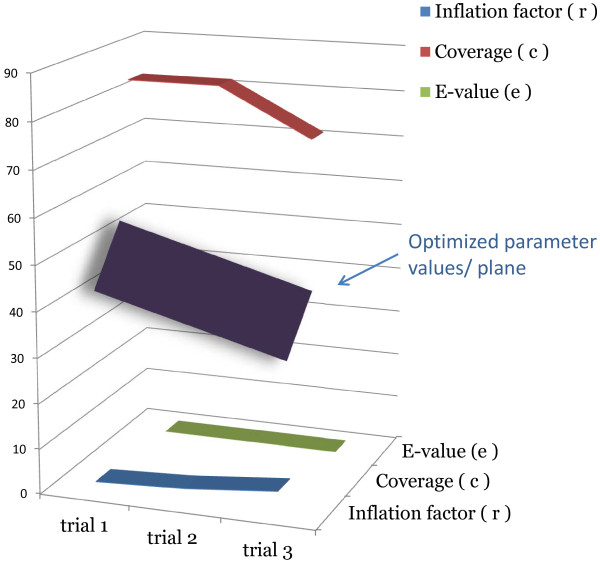
**Optimizing parameters for clustering.** Optimum values for the three parameters used for clustering with TribeMCL were chosen from the optimum plane consisting of the best possible combination of values (E-value, Inflation factor, and coverage).

Three parameters (E-value, inflation factor, and a new parameter called “coverage”) were evaluated and adjusted to optimize the performance [Additional file 2]. Coverage was defined as the percentage of the query sequences matched by sequences from the database, thus the higher the coverage is, the lower is the possibility of false-positives. We tested patterns of clustering with various combinations of parameters in the optimum plane of a three-parameter space [Figure 2] and settled on the following combination: E-value = 1e-50, inflation factor = 5, and coverage = 60%. The coverage parameter was instrumental in filtering out many false positives that display high E-values over short regions of similarity.

### CYP clustering in FCPD 1.2

Using the optimized parameters, we categorized 22,940 CYPs into 2,579 clusters (Figure 1): fungal and oomycete CYPs belong to 1,090 (42%) clusters, while the remaining clusters (1,489) contained only non-fungal CYPs. Although there are a few clusters that contain CYPs from more than one kingdom, most clusters are kingdom-specific. All oomycete clusters consist of CYPs in oomycete species with the exception of one that also contains CYPs in plants, fungi, and protists. Among the non-fungal clusters, 778 clusters contained plant CYPs and 652 clusters contained metazoan CYPs.

To validate our clustering approach and to link resulting clusters to results from previous classifications, the clusters were compared with CYP families and clans identified in previous studies [10,17], which in most instances showed good concordance between FCPD clusters and known families and clans. Out of 459 fungal CYP families identified in Nelson’s P450 databases, 292 matched with the CYPs in FCPD. Those that did not match corresponded to CYPs in species that are not currently covered in FCPD.

At the clan level, 77 clusters matched with 115 clans identified in a previous clan classification (Additional file 3) with some clusters including multiple clans. In only three instances our clustering results suggested that two or more clans needed to be merged: (i) clans CYP531 and CYP532; (ii) CYP619 and CYP530; and (iii) CYP567, CYP561, CYP563, and CYP60. Orphan clans identified in the previous classification [10] were assigned to some of the non-orphan clans through our clustering. We identified 38 new putative clans and validated existing clans, which brought the total number of clans in FCPD to 117.

As a result of this expanded clan classification, 131 additional CYP families were put into new and existing clans (Additional file 3). Of those, eight families that correspond to singlet FCPD clusters were classified as orphan clans. The resulting clans vary widely in size and number of CYP families included. The largest clans (CYP531 and CYP58) contain 14 families each. The size distribution analysis showed that, like many other protein families [25,26], CYP clusters follow a power law distribution (Additional file 4). Only 37 clusters with more than 100 members were observed. In contrast, 1,726 clusters were comprised of a single CYP. Information about individual clusters, families, and clans archived in FCPD will facilitate global analyses of fungal CYPs. New CYPs can be annotated using the BLAST search function.

### Wide variation of the CYPome

**Figure 3:**
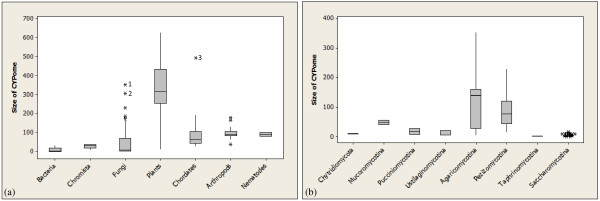
**Range of CYPome sizes across kingdoms and fungal phyla.** The boxplot shows the average number of CYPs across (**A**) kingdoms and (**B**) fungal phyla.

The total number of CYPs and their relative fraction within the total proteome in different kingdoms and phyla varied widely. The boxplots in Figure 3A show that plants have the largest CYPome (0.82%), bacteria have the smallest CYPome (0.05%), and fungi are placed in the middle (0.40%). The potato *Solanum phureja* has the largest CYPome composed of 629 CYPs.

The size of CYPome of individual species within kingdoms also varied drastically, presumably reflective of diverse lifestyles and ecologies. The largest variation was observed in fungi and plants. In fungi, Pezizomycotina and Basidiomycota have the largest and most variable CYPomes (Figure 3B). The CYPome of certain basidiomycota fungi such as the brown rot fungus *Postia placenta* (353 CYPs) and the cocoa tree pathogen *Moniliophthora perniciosa* (307 CYPs) are larger than typical plant CYPomes. In these species, massive expansions of CYPs involved in oxidizing complex hydrocarbons were observed [27]. In contrast, some basidiomycota fungi, such as *Puccinia graminis* (18 CYPs) and *Malassezia globosa* (6 CYPs), have undergone massive reductions, probably reflecting their obligatory pathogenic lifestyles. Members of the Chytridiomycota and Oomycota also showed small CYPomes. Members of Saccharomycotina and Taphrinomycotina have the smallest CYPomes among fungi (2–3 CYPs).

### Phyletic distribution of CYP families and clans in fungi

Our phyletic analysis showed an uneven distribution of CYP cluster sizes among taxa, which is consistent with extreme expansions and contractions of certain CYP families in the course of evolution. Seven out of the 30 largest fungal-specific clusters were exclusively composed of CYPs from the subphylum Pezizomycotina. The most dramatic expansions were observed in Pezizomycotina (clans CYP58 and CYP68), Agaricomycotina (clans CYP5150 and CYP63) and Mucoromycotina (family CYP509). Small clusters containing only species-specific CYPs were especially prevalent in members of Oomycota and Mucoromycotina.

The five largest fungal-specific clusters in FCPD had 1,056, 472, 452, 322, and 319 CYPs, respectively. These clusters represent some of the largest CYP families in fungi (Additional file 5, Additional file 6). The largest cluster (Cluster # 3) contains CYPs from the subphyla Agaricomycotina (Basidiomycota) and Pezizomycotina (Ascomycota). In this cluster, most Pezizomycotina CYPs (100) correspond to members of family CYP620, whereas 508 Agaricomycotina CYPs belong to family CYP5144. Some members of both families are known to be involved in xenobiotic metabolism [28]. Additionally, this cluster includes CYPs from the wood-rotting fungi *Heterobasidion annosum* (156) and *Postia placenta* (122), and more than 50 CYPs in six basidiomycete species, which suggests expansions of CYPs involved in the degradation of components of the wood (e.g., lignin, hemicellulose, cellulose).

The second largest fungal-specific cluster (# 11) has CYPs from Saccharomycotina and Pezizomycotina. It comprises the families CYP52, CYP548, CYP539, and CYP655 as well as a few other families involved in alkane assimilation (Additional file 7). The third largest cluster (# 12) consists of CYPs from Pezizomycotina. The most dominant family in this cluster is CYP65, which contains CYPs predicted to function in secondary metabolism.

Six clusters contain both fungal and non-fungal CYPs, many of which are involved in evolutionary conserved core metabolic roles and are likely derived from common ancestral proteins. Cluster 17 contains family CYP61, one of the most conserved CYP families in fungi and beyond. The cluster has CYPs from all sub-phyla of fungi, Amoebozoa, and the unicellular diatom *Capsaspora owczarzaki* as well as one CYP from the algae *Coccomyxa* sp. Cluster 22 includes families CYP505 and CYP541, and CYPs from all fungal taxa, Actinobacteria, Bacillariophyta, and the plant *Populus trichocarpa*. Cluster 7 includes CYPs from Zygomycota and Blastocladiomycota as well as oomycetes, protists, and plants. Cluster 8 includes a single family from the chytrid *Spizellomyces punctatus* and many CYPs from chordates. Cluster 13 contains members of CYP51, which are implicated in sterol biosynthesis in all fungal phyla [29], and various CYPs from Amoebozoa, Bacillariophyta, Euglenozoa, and Chordata. Lastly, cluster 69 contains CYP55 family, in which fungal and bacterial CYPs are clustered together. Some of these families will be discussed in more detail below.

**Figure 4:**
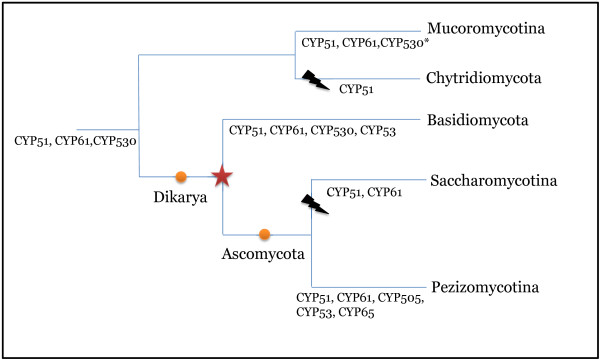
**Most conserved CYP families in fungi.** CYP families were compared across different sub-phyla to determine the most conserved CYPs. CYP51 and CYP61 were present in all fungi except a couple of chytrids.

Our clustering approach also revealed 959 phylum-specific clusters and 1,044 CYPs that did not belong to any previously defined CYP families. Out of these, 560 were present in singlet clusters. CYP families present in individual phyla and subphyla (excluding Saccharomycotina) were also examined. Five CYP families were present in all species from Pezizomycotina and four families were present in all basidiomycete species, while 10 families were present in all species from Mucoromycotina. Among them, two families (CYP51 and CYP61) were common to all taxa. The CYP530 family is absent in the ascomycota fungi, however all the other basal lineages have retained this family (Figure 4). The most parsimonious explanation is that CYP51, CYP61, and CYP530 were present in the last common ancestor of all fungi. Indeed, CYP51 is thought to be present even in early eukaryotes, and it has been hypothesized that CYP61 evolved from CYP51 [30]. On the other hand, the family CYP530 seems to be specific to fungi and is known to be involved in degradation of various fatty acids and hydrocarbons (Additional file 8: xenobiotic metabolism), allowing fungi to utilize these materials as nutrient sources. 

### Functional annotation and classification of CYP clusters

To assign putative functional roles to individual clusters, we conducted a comprehensive literature review for functionally characterized fungal CYPs. This survey led to the identification of 54 CYPs that had been shown to be involved in (i) primary metabolism (15 CYPs), (ii) secondary metabolism (28) or (iii) xenobiotic metabolism (11) (Additional file 5). We then used BLASTp to search the FCPD database with these CYPs as queries (Methods). A total of 2,457 hits (E-value cutoff of 1e-100) were generated with the CYPs involved in various primary metabolic reactions. This high number of hits is mainly due to the presence of well-conserved, house-keeping families such as CYP51 and CYP61, which are involved in ergosterol biosynthesis [29,30]. Additionally, we found 544 and 642 hits with those CYPs involved in secondary and xenobiotic metabolism, respectively (Additional file 9). Only one family (CYP58) contained CYPs involved in both secondary and xenobiotic metabolism. For instance, one such CYP58 gene from *Phanerochaete chrysosporium* has been predicted to function as benzoate 4-hydroxylase (xenobiotic metabolism) and at the same time is also involved in trichothecene biosynthesis (secondary metabolism) [31]. The relatively small number of hits to CYPs involved in secondary metabolism suggests that many fungi might have evolved a lineage-specific repertoire of CYPs to produce specific secondary metabolites.

Excluding CYP58, we found 12, 30, and 12 CYP families that uniquely matched to the primary, secondary, and xenobiotic metabolism categories, respectively. These 54 CYP families were then used to assign putative functional roles to the respective clans. With this approach we tentatively classified a total of 34 clans into primary (5 clans), secondary (17), and xenobiotic (12) metabolism (Additional file 8).

### Detailed analysis of specific clans

Selected CYP clans and families were analyzed in detail to augment and validate previous evolutionary studies [28-34] and to uncover notable features.

### Clans 51 and 61

Our clustering analysis fully supported families CYP51 and CYP61, which are composed of house-keeping CYPs found in almost all fungi, plants and animals. CYP51 is a lanosterol 14-alpha demethylase involved in 14-demethylation of sterol precursors, and this demethylation step is common throughout all organisms [35]. To better understand its evolution, we constructed a phylogenetic tree with members of CYP51s from fungi, the early opisthokonts and other single-celled eukaryotes (Additional file 10).

Most yeast species have a single CYP51 gene, whereas most Pezizomycotina species have two genes with the exception of *Fusarium* species and *Aspergillus carbonarius* (three genes). Basidiomycetes also have a single gene with the exception of *Postia placenta* and *Coprinus cinereus* (two genes). *Rhizopus oryzae*, *Allomyces macrogynus*, and *Fragilariopsis cylindrus* have two CYP51 genes and no CYP61 genes. This is consistent with the view that the CYP51 gene became duplicated very early in fungal evolution and then one of the duplicates may have given rise to CYP61 [30].

CYP61 gene is a 22 sterol desaturase that carries out one of the last reactions in the Ergosterol metabolism pathway. The phylogenetic analysis of CYP61 (Additional file 11) revealed the presence of a single gene in all yeasts and all basidiomycetes except *P. placenta* (two genes). Most ascomycota species have at least two genes with the exception of *Pneumocystis carinii*, as well as the basidiomycetes *Puccinia graminis*, and *Melampsora laricis-populina*, all three of which do not have a CYP61 gene. The absence of CYP61 genes in these species could be due to their obligate lifestyle, wherein they may utilize essential sterols from the plant/animal hosts.

### Clans 65 and 68

Clans CYP65 and CYP68 consist of CYPs that belong to the secondary metabolism category. CYP65 has been found to catalyze the epoxidation reaction during the biosynthesis of the mycotoxin trichothecene, as well as during radicicol biosynthesis (Additional file 5, Additional file 12 and Additional file 13). CYP68 carries out the C-8 oxygenation reaction during trichothecene biosynthesis ( Additional file 5, Additional file 14) and the oxidation reaction during the biosynthesis of the plant hormone gibberellin [36]. The phylogenetic trees of CYP65 and CYP68 reveal multiple recent duplications and expansions (Additional file 12, Additional file 13 and Additional file 14). These clans are absent in ascomycete yeasts and basidiomycete species, suggesting that they might have emerged in the ancestor of the Pezizomycotina.

Among members of the Pezizomycotina, there is a wide variation in the number of CYPs in clans CYP65 and CYP68. The *Coccidioides* species have just one CYP65 gene, whereas Dothideomycetes and *Aspergillus* species have 8–10 genes for CYP65s and 3–4 genes for CYP68s. Dothideomycetes have on average at least 5–6 more genes than other fungi, which is consistent with their secretion of diverse host-selective toxins (HSTs, [37]). Many of these HSTs are products of secondary metabolism pathways.

The highest number of CYP65 and CYP68 clan members is seen in *Magnaporthe oryzae*, *Colletotrichum graminicola* and *Colletotrichum higginsianum* (Additional file 12 and Additional file 14). All three fungi form appresoria (specialized infection structures formed by germinating spores) to enter the plant cell. Expression studies have demonstrated that secondary metabolism pathways are active during the infection process [38], suggesting that the increased number of CYP65 and CYP68 family members in these fungi might be linked to their pathogenicity.

### Clan 505

CYP505 members are fatty acid hydroxylases that carry out the subterminal omega hydroxylation of fatty acids, a step required for the use of fatty acids as an energy source. It was hypothesized that CYP505 in fungi has evolved from the bacterial CYP450BM3 via a horizontal gene transfer (HGT) event [32]. This hypothesis is supported by the fact that both types have a fused NADPH CPR domain ( http://drnelson.uthsc.edu/P4503d.html).

To test this HGT hypothesis, we performed a phylogenetic analysis of this clan (includes 161 CYPs from families CYP505 and CYP541). Contrary to the hypothesis, the tree topology (Additional file 15) suggests an ancient origin of this clan in eukaryotes and subsequent losses in certain lineages. The earliest members of the clan seem to be present in the unicellular opisthokonts *Capsaspora owczarzaki*, Streptomyces species of bacteria and the unicellular algae *Fragilariopsis cylindrus*. There are at least two genes for CYP505 in most fungi, while early eukaryotes *F. cylindrus* and *Allomyces macrogynus* have 4–5 genes, suggesting an early increase in its copy number and subsequent gene losses. CYP505s are absent in ascomycete yeasts. Among members of the Pezizomycotina, *A. flavus* and *Podospora anserina* have five genes, and *M. grisea* has four genes. Basidiomycetes also have at least two genes with the white rot fungus *P. chrysosporium* containing six genes. It has been hypothesized [39] that CYP505 is used by plant-associated fungi to degrade plant cuticle which is synthesized by in-chain hydroxylation of fatty acids [40].

### Clan 52

Cluster 11 contained all the CYPs belonging to clan CYP52. The highest numbers of CYP52 proteins (12) are seen in *Aspergillus flavus*, *A. niger* CBS 513.88, *Trichoderma virens* Gv29-8, *Botrytis cinerea* and *Magnaporthe oryzae*. *Talaromyces stipitatus* and *Penicillium marneffei* have 10 and 11 members of CYP52, respectively. In *M. oryzae*, CYP52 is upregulated during the penetration of the plant cuticle, which is made up of hydrocarbons [41]. Similar processes could be happening in *B. cinerea* and *A. flavus*, both of which are pathogenic to plants as well as *Trichoderma virens* Gv29-8 (twelve genes), *T. reesei* (nine genes), and *T. atroviride* (six genes) that are known to penetrate the fungal cell wall [42] as well as plant roots [43]. CYP52 genes are found in *Candida* species that are known to metabolize alkanes and other hydrocarbons, but are absent in *Saccharomyces cerevisiae* and *Schizosaccharomyces pombe*[44]. There were as many as 12 CYP52 proteins encoded by *Yarrowia lipolytica*, but there were no CYP52 proteins in basidiomycetes. All of these species might be using their CYP52 repertoire to support these processes, and expansion of the CYP52 family in these ascomycete fungi may allow efficient metabolism of various hydrocarbon compounds. We built a neighbor-joining tree to look at their evolutionary relationships (Additional file 16). The most parsimonious evolutionary scenario suggests that the family evolved in the ancestor of budding yeasts but was lost in the lineage including *S. cerevisiae* but then expanded in the Pezizomycotina.

### Clan 53 and Clan 504

CYP53 is a benzoate-para-hydroxylase enzyme that was first discovered in *Aspergillus niger*[45]. This benzoate detoxification occurs via the beta-ketoadipate pathway [46], which is present in many soil microbes that degrade aromatic compounds, some of which are released by plants [47]. Although benzoate detoxification appears to be the main function of members of this CYP group, some of them have also been found to exhibit O-demethylation activity [28]. Clan 53 is a single family clan in cluster 37 and contains 89 CYPs. This family is absent in ascomycete yeasts. A wide variation in its size was observed in the wood-decaying fungi *Postia placenta* (14 genes), *Pleurotus osteratus* (three genes) and *Phanerochaete chrysosporium* (one gene). Considering their proposed role in degrading plant-based aromatic compounds that are released by the plants into the soil or that might be present as a part of the dead plant material, this wide variation is puzzling. They are also present in several plant-pathogenic fungi such as *Fusarium oxysporum* (3), *F. graminearum* (4), *Puccinia graminis* (1), *Moniliophthora perniciosa* (2), *Cochliobolus heterostrophus* (3), and *Botrytis cinerea* (2), suggesting the possibility that the benzoate degrading activity may contribute to pathogenesis.

Clan CYP504 includes CYPs that are involved in phenylacetate catabolism [48]. Specifically, they are involved in the ortho-hydroxylation of phenylacetate, which is a precursor in penicillin production. Like Clan 53, this clan is a single-family cluster (cluster 29; Additional file 17). The family is found in many saprophytic species as well as a number of basidiomycetes fungi that can degrade phenol derivatives as a source of carbon [49]. This family is also present in a number of human and plant-pathogenic fungi like *Stagonospora nodorum* (three genes), *C. heterostrophus* (four genes), *Penicillium marneffei* (five genes), *Fusarium oxysporum* (three genes), *F. graminearum* (four) and *F. solani* (five genes). Both CYP53 and CYP504 family members were found to be upregulated during cuticle infection by insect pathogenic fungi *Metarhizium anisopliae* (four genes) and *M. acridum* (two genes) [50]. It was suggested that in these insect pathogens these CYP families carry out detoxification of insect released phenylacetate [50,51].

### Clan 533

This clan forms one of the largest fungal clusters. It contains 15 CYP families; two of them are specific to the Ascomycota, 10 are specific to the Basidiomycota, and three (CYP533, CYP620 and CYP621) are common to both. The three common families form clan 533 in the previous classification by Deng et al. [10]. CYPs belonging to the CYP533 family seem to be involved in secondary metabolism since they show similarity to CYPs involved in the biosynthesis of sterigmatocystin and aflatoxin. The largest basidiomycete-specific family in this clan is the CYP5144 family that has 354 members, some of which have been found to be involved in the degradation of polyaromatic hydrocarbons (PAH) [28]. Many CYPs in this cluster exist in the brown rot fungus *Postia placenta* (120 CYPs), the forest pathogen *Heterobasidion annosum* (78 CYPs), the mushrooms *Coprinus cinereus* (61) and *Pleurotus osteratus* (60), the white rot fungus *Phanerochaete chrysosporium* (56), and the dry rot fungus *Serpula lacrymans* (55). Among ascomycetes, *Aspergillus flavus* (8), *A. oryzae* (8), *A. niger* (5), *Fusarium verticillioides* (6), *F. oxysporum* (7), *F. graminearum* (7), and *Trichoderma virens* (5), all of which are known for their capability of producing various secondary metabolites, have the largest numbers of CYP5144 members. The presence of CYP5144 (PAH and xenobiotics degradation) and CYP533 (secondary metabolite biosynthesis) in this cluster indicate that these families might have evolved from a common ancestral CYP family.

### CYPs in Mucoromycotina, Blastocladiomycota and Oomycota

Most CYPs from Mucoromycotina, Blastocladiomycota and Oomycota clustered separately into taxa-specific clusters. CYPs from Mucoromycotina were divided into 28 clusters, which include three clusters that also included non-fungal CYPs (CYP51, CYP61, and CYP505) and 22 clusters only having Mucoromycotina CYPs. One of the clusters (# 7) had CYPs from Mucoromycotina as well as CYPs from Oomycota, Blastocladiomycota, protists, plants, and *Ustilago maydis* (Basidiomycota). Plant CYPs in this cluster (belonging to clan CYP86) included enzymes shown to modify fatty acid and alkane substrates. This pattern suggests a very ancient origin of this alkane metabolizing CYP clan, potentially predating the split of the eukaryotes into Unikonts, Plantae and Chromalveolates. In this scenario, this family might have been lost in most fungi. Lateral transfer of the CYP family from plants to early fungi could be another possibility, especially considering the narrow distribution of these CYPs in fungi at the ancestral nodes of the fungal species tree. Lastly, it is also possible that convergent evolution could have driven the CYPs to perform similar functions in both plants and fungi. Blastocladiomycota CYPs also exhibited a pattern similar to those seen in Mucoromycotina. Only three clusters contain CYPs from other phyla. Interestingly, there are no CYP61s in Blastocladiomycota, possibly indicating their loss of ability to synthesize ergosterol. Fourteen clusters contain only Blastocladiomycota CYPs. Most CYPs from Mucoromycotina and Blastocladiomycota exhibited low similarity to CYPs in Nelson’s P450 databases.

As expected, oomycota CYPs mostly formed oomycete-specific clusters (18) with the exception of cluster 7, which also contains CYPs from Basidiomycota, Zygomycota (in fungi) and plants. There are 11 CYPs that do not show any significant similarity to CYPs in Nelson’s P450 databases. Only four known CYP families (CYP5014-5017) were identified. Members of CYP5015 (30) showed 30% identity (89% coverage) to CYP94 in *Arabidopsis thaliana*, which is involved in fatty acid metabolism. Similarly, those in CYP5014 (37) showed 34% identity (89% coverage) to fatty acid omega hydroxylases (CYP86) in *Medicago truncatula*. Members of CYP5016 (5) and CYP5017 (8) also showed similar levels of identity to fatty acid hydroxylases. Thus, most CYPs in oomycete species, which encode about 30–40 CYPs, could be involved in fatty acid metabolism. Our observations are consistent with previous studies that predicted the absence of extensive secondary metabolism clusters (and consequently CYPs) in oomycetes [9,52].

### CYPs with unusual phyletic profiles

Analysis of several clusters that contained CYPs from more than one kingdom revealed patterns suggesting rapid birth–death evolution, or alternatively, horizontal gene transfer (HGT), which has been implicated as a contributing factor in fungal adaptation to new ecological niches [53-58]. However, in most cases, due to low taxon sampling, it would be premature to make any firm conclusions.

Our analysis of clusters 23 and 69 exhibited patterns similar to previously published examples of HGT in *Fusarium oxysporum*[32] and *Phanerochaete chrysosporium*[59]. Cluster 69 contains CYP55s from *P. chrysosporium*, Pezizomycotina, and the bacterial genus *Streptomyces*. Similarly, cluster 23 (clan CYP505) contains CYPs from bacteria, plants, early opisthokonts, and fungi. Cluster 46 has 72 CYP540 members including five CYPs of Mucoromycotina species that show high sequence similarity to bacterial CYPs. Phylogenetic analysis showed two branches, one with only fungal CYPs and the other with bacterial and Mucoromycotina CYPs (Additional file 18), which suggests the possibility of lateral gene transfer.

Clan CYP5081 (Cluster 126) is composed of 18 intron-less CYPs including four from *Aspergillus* spp. and three from *Microsporum* spp. The CYPs from *A. fumigatus* were predicted to be involved in helvolic acid biosynthesis [60], and their orthologs in the insect pathogens *Metarhizium anisopliae* and *M. acridum* are expressed during cuticle infection [61]. The observed phyletic pattern is consistent with massive gene loss in most fungi, but HGT from nitrogen-fixing bacteria that also synthesize helvolic acid [60] cannot be completely excluded.

Clan CYP544 (Cluster 109) contains 21 CYPs mainly from plant pathogens and epiphytes (fungi that survive on the surface of plants). Some members share sequence similarity with CYPs involved in the biosynthesis of camptothecin [62], an alkaloid secreted by plants that have anti-cancer properties. This cluster includes two homologs from *Fusarium solani*, with one of them identified as a pseudoparalog [63]. This pseudoparalog lies on a dispensable chromosome in *F. solani*, and shows similarity to CYP94 family members from plants [64]. Other CYPs in the cluster also show similarity to plant CYPs belonging to clan CYP86. Our phylogenetic analysis ( Additional file 19) suggests potential HGT from plants to fungi intimately associated with plants [62].

We also analyzed clusters 173 and 212, which contain 10 and 7 CYPs from plant-pathogenic and plant-associated fungi, respectively. While Cluster 173 has CYPs from four different basidiomycota fungi, Cluster 212 has seven CYPs from *Puccinia graminis*. All the CYPs in these clusters belong to families CYP5025 and CYP5026, respectively and share significant similarity to CYP86 and CYP704, families that are involved in the metabolism of complex hydrocarbons such as fatty acids and in the biosynthesis of plant cutin [65]. The phylogenetic analysis (Additional file 20) suggests that clan CYP86 in plants and families CYP5025/CYP5026 in fungi have arisen from a common ancestral CYP family.

Finally, three CYPs from *Fusarium* species (Cluster 416, Clan CYP645) showed sequence similarity to bacterial P450RhF proteins [66]. The RhF CYPs represent the first known example of bacterial CYPs that receive electrons from a FMN- and Fe/S- reductase fused to them [67]. No other fungus has been observed to have this type of CYP. The result of our phylogenetic analysis (Additional file 21) is consistent with the presence of this type of CYP in the ancestor of *F. oxysporum* and *F. graminearum*.

## Conclusion

Here we present a new version of FCPD, which holds 9,697 CYPs from 113 fungal and oomycete species in addition to CYPs from selected species in other kingdoms. There is no perfect solution to clustering proteins as diverse and numerous as CYPs, but we believe that our clustering pipeline provides an improved CYP classification system. Using this pipeline we have identified new clans and families. To our knowledge, this study represents the most extensive classification of fungal and oomycete CYPs, which will facilitate functional annotation and classification of putative CYPs encoded by newly sequenced fungal and oomycete genomes. The FCPD 1.2 pipeline can efficiently group CYPs from newly sequenced genomes and help predict their functions.

The CYP number for certain species may have been exaggerated due to the following factors: (i) heterozygous alleles of the same gene, and (ii) artifacts created during genome assembly and annotation being counted as unique genes. Some species are diploids with certain degrees of heterozygosity between alleles, which might have been counted as unique genes, thus increasing the total number of CYPs. In some cases gene fragments (arising from errors during genome assembly) have been counted as separate genes. Rectifying these potential artifacts manually is challenging due in part to the very large size of data present in FCPD and also due to the difficulties of validating individual data.

There is also CYP redundancy in the database due to the presence of CYP sequences from multiple strains of several species. In the case of *Postia placenta*, which encodes the largest CYPome among fungi, we identified eight alleles that have been counted as separate genes. Similar analysis of the *Solanum phureja* CYPome (the largest among plants) showed four alleles that had been identified as distinct genes. Because the database includes data from 112 strains from 26 species, there is redundancy in the CYP data. We caution that users should keep these caveats in mind when using the database.

Our analysis of fungal CYPs points to a number of notable evolutionary patterns. Gene duplication and subsequent modification of the duplicated copies seem to have played a major role in creating the observed CYP diversity. The CYP family expansions seen in some of the basidiomycetes like *Postia placenta*, *Heterobasidion annosum*, and *Phanerochaete chrysosporium* as well as ascomycetes such as *Magnaporthe oryzae*, *Stagonospora nodorum*, *Fusarium solani*, and *F. oxysporum* may have led to these fungi adapting to their current ecological niches. Although massive CYP gene losses probably underpin unusual phyletic profiles, horizontal gene transfer as a mechanism cannot be completely discounted. The curated CYP dataset in FCPD 1.2 provides a solid foundation for in-depth studies on myriad evolutionary patterns, which will contribute to understanding fungal evolution.

## Methods

### Acquisition of data and phylogenetic analyses

In total, 323 genomes stored in the Comparative Fungal Genomics Platform (CFGP) [12] were used to identify CYPs. Sixteen Interpro domains associated with CYP proteins were used to identify CYPs. To filter out false positives, domains that spanned fewer than 25 amino acids were labeled as “questionable” and manually evaluated as previously described [22]. The filtered sets of protein sequences were used for clustering (Additional file 1).

Phylogenetic analyses were performed using the neighbor-joining (NJ), minimum evolution (ME), and maximum-likelihood (ML) methods as implemented in MEGA version 5.05 with 1,000 bootstraps [68]. In order to deal with alignment gaps we used a pair-wise deletion method for NJ and ME trees, whereas complete deletion was used in building ML trees. Default parameter values were used for all the phylogenetic methods. The alignments were constructed with ClustalW option of MEGA, with Gonnet matrix and default parameter values. In each case, the most prevalent phylogenetic tree with the best bootstrap support was chosen for further analysis. In some cases, such as Additional file 18, Additional file 19, Additional file 20, and Additional file 21, phylogenetic trees were built with GenBank sequences extracted via Blast with selected CYP queries. This was done to include CYPs from species that were not represented in the FCPD.

### Clustering of the CYPs using BLASTp and the optimized Tribe-MCL algorithm

CYP sequences were clustered using the optimized Tribe-MCL algorithm [21]. Reciprocal Blast searches were performed to identify putative ortholog groups to be submitted to the clustering algorithm. The Tribe-MCL clustering procedure is dictated by two main parameters: (i) E-value obtained from the pair-wise BLASTp comparison of all CYPs (default value 1e-5 or lower) and (ii) the inflation factor (indicating “tightness” of the cluster) at the highest value 5 [15]. To improve the classification, we added one more parameter, “coverage”, which was defined as the percentage of the query sequences matched by sequences from the database. To find optimal conditions for these three parameters, we tested efficiency of clustering with various combinations: (i) e-values between 1e-10 and 1e-100 at intervals of 1e-10; (ii) nine coverage values from 20% to 100% at intervals of 10%, and (iii) inflation factor from 1 to 5. We empirically chose optimal parameters as: e-value = 1e-50, coverage = 60%, and inflation factor = 5 (Additional file 2).

### Clan identification

We were able to expand the clans identified in earlier studies [10,31,69] through our optimized clustering procedure. We searched for each clan through our database using a search function that was built to facilitate searching the database using various terms (e.g., Sequence ID, taxonomic group, and CYP family). We followed this step for all the clans mentioned in previous studies [10,17,31,69], which allowed us to identify novel clans and assign CYP families to previously identified orphan clans (Additional file 3). There were a number of CYPs that did not show any significant similarity to any of the CYP families in Nelson’s P450 databases, indicating that they are members of novel CYP families. Most of them were present in singlet clusters.

### Classification of CYPs into putative functional categories

An extensive literature search was performed to identify 54 functionally characterized fungal CYPs. These CYPs were then matched to CYPs in FCPD using BLASTp with an E-value cutoff of 1e-100. This stringent E-value was chosen based on an empirical testing of several E-values. Based on similarity to the characterized CYPs, CYP families were classified into three broad functional categories: (i) primary metabolism, (ii) secondary metabolism, and (iii) xenobiotic metabolism. Many of the hits occurred in more than one category. In order to link CYP clans into these functional categories, we have transferred functional annotations described above into respective clans. The BLASTp hits and the characterized set of CYPs can be accessed at http://p450.riceblast.snu.ac.kr/char_p450.php.

### Online database architecture

FCPD has been developed using PHP script with MySQL database [22]. The Linux-based apache web-server and task management system supports BLAST analysis and MCL clustering. The middle-ware written in Perl script simultaneously executes the bioinformatics pipelines from the query submitted by the end-user, and retrieves the archived CYP dataset. The pipeline for FCPD can be found in Additional file 1.

## Competing interests

The authors declare that they have no competing interests.

## Authors’ contributions

VPM wrote the draft manuscript, designed the pipeline and carried out all the detailed analyses. JP set up the database and designed the pipeline. BP and JC contributed to database improvement and data curation. NF-A co-wrote the manuscript. SK and Y-HL conceptualized and coordinated the study and guided manuscript preparation. All authors read and approved the final manuscript.

## Supplementary Material

Additional file 1 **Pipeline employed in FCPD 1.2 version.** The pipeline still consists of four steps employed in building the previous version of FCPD, but step 3 is now based on optimized parameters. Additionally, a new parameter, coverage, was added to the clustering procedure to further improve clustering results. (PDF 235 kb) Click here for file

Additional file 2 **Parameter optimization for clustering.** (XLSX 10 kb) Click here for file

Additional file 3 **Distribution of CYP families into clans.** (DOCX 26 kb) Click here for file

Additional file 4 **CYP family sizes follow a power law distribution.** The graph shows the family size distribution across families. (PDF 167 kb) Click here for file

Additional file 5 **Characterized CYPs used for functional classification.** (XLSX 12 kb) Click here for file

Additional file 6 **Top 10 CYP families in fungi.** (XLSX 8 kb) Click here for file

Additional file 7 **The 30 largest clusters containing only fungal and oomycete CYPs.** (XLSX 10 kb) Click here for file

Additional file 8 **Clans involved in the primary, secondary and xenobiotic metabolisms.** (XLSX 9 kb) Click here for file

Additional file 9 **Blast hits to characterized CYPs.** (XLSX 10 kb) Click here for file

Additional file 10 **Neighbor joining tree of CYP51.** (PDF 703 kb) Click here for file

Additional file 11 **Neighbor joining tree of CYP61.** (PDF 552 kb) Click here for file

Additional file 12 **Phylogenetic tree of CYP65 in Pezizomycotina.** Basidiomycetes and Ascomycete yeast species lack family CYP65. In Pezizomycotina, there is large variation in the number of CYP65 family genes. *Coccidioides* and *Neurospora* spp. have only one and two members, respectively. On the other hand, Dothideomycetes fungi have 6–15 members. The tree was adapted from Medina et al. [70]. (TIFF 7901 kb) Click here for file

Additional file 13 **Neighbor joining tree of CYP65.** (PDF 822 kb) Click here for file

Additional file 14 **Phylogenetic tree of CYP68.** CYP68 family members are found in a number of secondary metabolism gene clusters. This family was lost in yeasts, and is absent in most Basidiomycetes except for some Homobasidiomycetes species. The tree was adapted from Medina et al. [70]. (TIFF 8118 kb) Click here for file

Additional file 15 **Neighbor joining tree of CYP505-CYP541.** (PDF 507 kb) Click here for file

Additional file 16 **Maximum-likelihood tree of CYP52.** (PDF 431 kb) Click here for file

Additional file 17 **Maximum-likelihood tree of CYP504.** (PDF 395 kb) Click here for file

Additional file 18 **Phylogenetic tree of CYP540.** (PDF 548 kb) Click here for file

Additional file 19 **Phylogenetic tree of CYP544.** (PDF 443 kb) Click here for file

Additional file 20 **Phylogenetic of CYP5025.** (PDF 437 kb) Click here for file

Additional file 21 **Phylogenetic of CYP645.** (PDF 354 kb) Click here for file
