# Supplementary material for: Systematic and searchable classification of cytochrome P450 proteins encoded by fungal and oomycete genomes
Source: BMC Genomics. 2012 Oct 4;13:525. doi: 10.1186/1471-2164-13-525 (PMC3505482; doi:10.1186/1471-2164-13-525)
Supplement: Additional file 1 — Pipeline employed in FCPD 1.2 version. The pipeline still consists of four steps employed in building the previous version of FCPD, but step 3 is now based on optimized parameters. Additionally, a new parameter, coverage, was added to the clustering procedure to further improve clustering results. (PDF 235 kb) [file 1471-2164-13-525-S1.pdf]

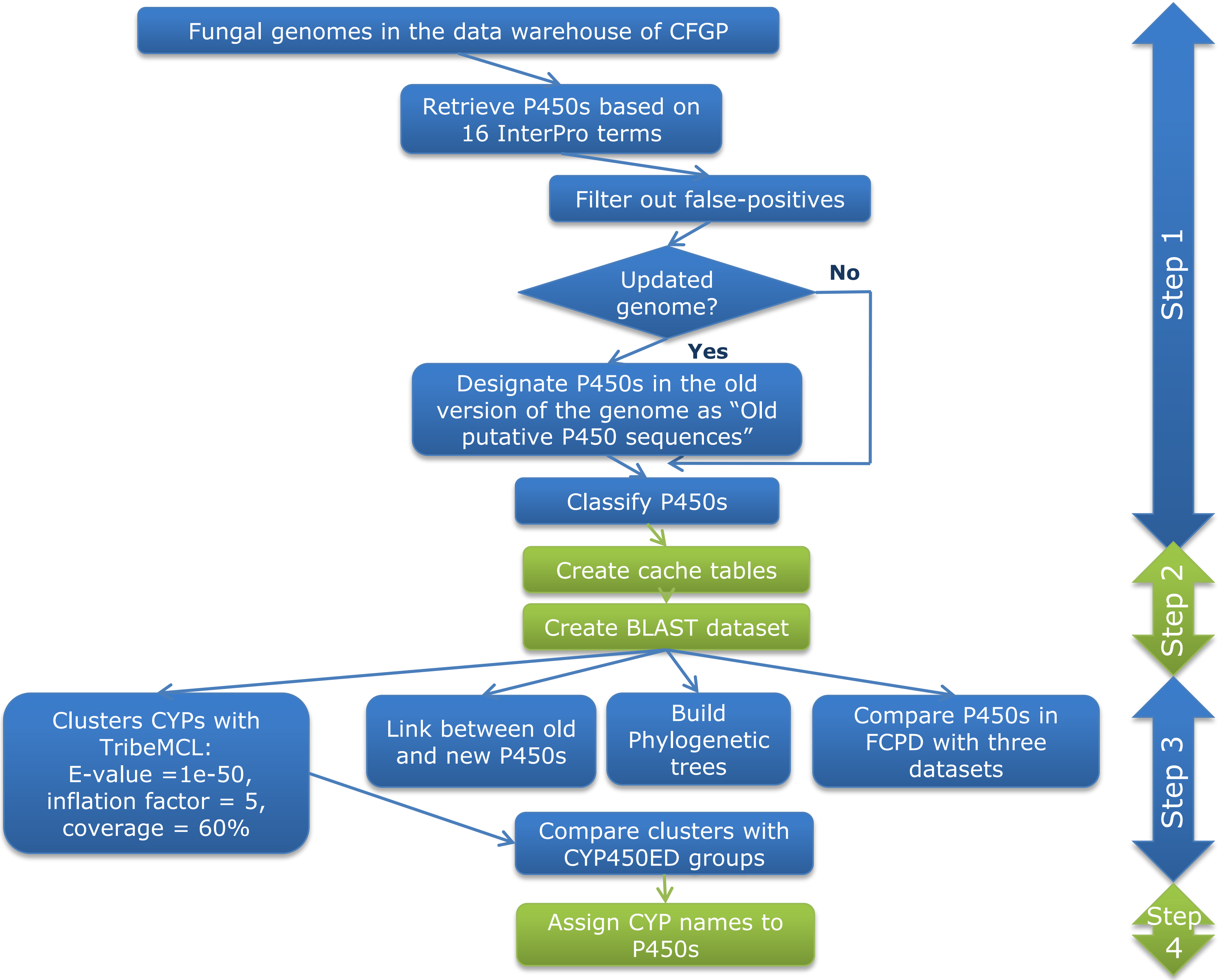

Pipeline employed in the FCPD 1.2 version

The pipeline still consists of four steps employed in building the previous version of FCPD, but step 3 is now based on optimized parameters. Additionally, a new parameter, coverage, was added to the clustering procedure to further improve clustering results.
