# Supplementary material for: Systematic and searchable classification of cytochrome P450 proteins encoded by fungal and oomycete genomes
Source: BMC Genomics. 2012 Oct 4;13:525. doi: 10.1186/1471-2164-13-525 (PMC3505482; doi:10.1186/1471-2164-13-525)
Supplement: Additional file 3 — Distribution of CYP families into clans. (DOCX 26 kb) [file 1471-2164-13-525-S3.docx]

**CYP families and clans**

| **Clans** | **Families** |
| --- | --- |
| 51 | 51 |
| 52 | 52, 538, 539, 584, 585, 655, **5087, 5113, 656, 5203** |
| 53 | 53 |
| 54 | 54, 503, 560, 599, 602, 604, 649, 5204, 5213, **5085, 5086, 5103, 601** |
| 55 | 55 |
| 56 | 56, 661, 509, 5210, 5211, 5212, **5099** |
| 58 | 58, 542, 551, 552, 681, 682, **580, 680, 579, 5094, 5095, 5096, 5105, 5112** |
| 59 | 59, 586, 587, 662 |
| 61 | 61 |
| 62 | 62, **684, 626** |
| 63 | 63 |
| 64 | 5206, 5207, 5208, 5209 |
| 65 | 65, 561, 562, 563, 564, 565, 567, 568, 5117, 5118, 60 |
| 68 | 68, 595, 596, 622, 650, 651, 652, **597, 598, 5061, 5067, 5073, 5074** |
| 504 | 504 |
| 505 | 505, 541, 5205 |
| 506 | 506 |
| 507 | 507, 527, 535, 570 |
| 512 | 512 |
| 526 | 526, 591, **638**, 644 |
| 528 | 528 |
| 529 | 529, 543, 545, **592** |
| 530 | 530, **5027, 5065, 5066, 5068, 5069, 5148, 619, 663,665, 5093,5098,5119** |
| 531 | 531, 631, 532, 57, 536, 629, 674, 675, 676, **5028, 5077, 5078, 5080, 5104** |
| 533 | 533, **502**, 620,621,**64,5037,5144,5145,5146,5147,5149,5152** |
| 534 | 534 |
| 537 | 537, 577 |
| 540 | 540 |
| 544 | 544 |
| 546 | 546, **5053** |
| 547 | 547, 581, 582, 616, 617, 618, **5070** |
| 548 | 548, **5114, 5115** |
| **549** | **549** |
| 550 | 550, 553, 633, 634, 635, 636, 660, **610, 611, 612** |
| 559 | 559, 606, 623, 647 |
| 566 | 566 |
| 572 | 572, 573, **5109** |
| 574 | 574, **5029, 5076**, 628, 669, 670, 671, |
| **575** | **575** |
| **576** | **576** |
| 578 | 578, **625** |
| **589** | **589, 5075, 614** |
| **590** | **590** |
| 593 | 593 |
| 603 | 603 |
| 605 | 605 |
| **607** | **607** |
| **608** | **608** |
| **609** | **609** |
| 613 | 613, 686, **685, 5082** |
| **615** | **615** |
| **624** | **624** |
| **627** | **627, 5030** |
| 630 | 630 |
| **632** | **632** |
| **637** | **637** |
| **639** | **639, 5100** |
| **640** | **640** |
| **642** | **642** |
| 643 | 643 |
| **645** | **645** |
| **646** | **646** |
| **648** | **648** |
| 653 | 653, 654 |
| **657** | **657, 641** |
| **659** | **659, 5090, 5111** |
| **664** | **664** |
| **666** | **666** |
| **667** | **667** |
| **672** | **672** |
| **673** | **673** |
| **677** | **677, 5064, 5142** |
| **678** | **678** |
| **683** | **683** |
| **687** | **687** |
| **698** | **698** |
| **5014** | **5014, 5015** |
| **5016** | **5016** |
| **5017** | **5017** |
| **5025** | **5025, 5026** |
| **5031** | **5031** |
| **5032** | **5032** |
| **5035** | **5035, 5036** |
| **5042** | **5042** |
| **5052** | **5052** |
| **5058** | **5058** |
| **5063** | **5063** |
| **5071** | **5071, 5106** |
| **5081** | **5081** |
| **5083** | **5083** |
| **5084** | **5084, 5121** |
| **5089** | **5089** |
| **5091** | **5091** |
| **5092** | **5092** |
| **5097** | **5097** |
| **5101** | **5101** |
| **5102** | **5102** |
| **5108** | **5108** |
| **5110** | **5110** |
| **5116** | **5116** |
| **5136** | **5136, 5137** |
| **5139** | **5139, 5151, 5034, 5033, 5138** |
| **5140** | **5140** |
| **5141** | **5141, 5154** |
| **5143** | **5143** |
| **5150** | **5150, 5155** |
| **5153** | **5153** |
| **5156** | **5156** |
| **5157** | **5157** |

There were certain families that were found in singlet clusters (containing a single CYP), these were left as orphan CYP clans: CYP511, CYP67, CYP583, 658, 668, 679, 5120, and 5125

The following CYP families from Nelson’s database did not have matches in FCPD 1.2: CYP5160-CYP5190, 5200-5400, CYP6000, CYP501, CYP5038, CYP5039, CYP5040, CYP5043, CYP5044, CYP5045, CYP5046, CYP5047, CYP5048, CYP5049, CYP5050, CYP5051, CYP5054, CYP5055, CYP5056, CYP5057, CYP5060, CYP5062, CYP510, CYP5107, CYP5127, CYP5128, CYP5129, CYP5130, CYP5131, CYP5132, CYP5133, CYP5134, CYP5135, CYP5159, CYP557, CYP5667, CYP66, CYP69, CYP697 and CYP699**.**
