# Supplementary figures and images for: Systematic and searchable classification of cytochrome P450 proteins encoded by fungal and oomycete genomes
Source: BMC Genomics. 2012 Oct 4;13:525. doi: 10.1186/1471-2164-13-525 (PMC3505482; doi:10.1186/1471-2164-13-525)

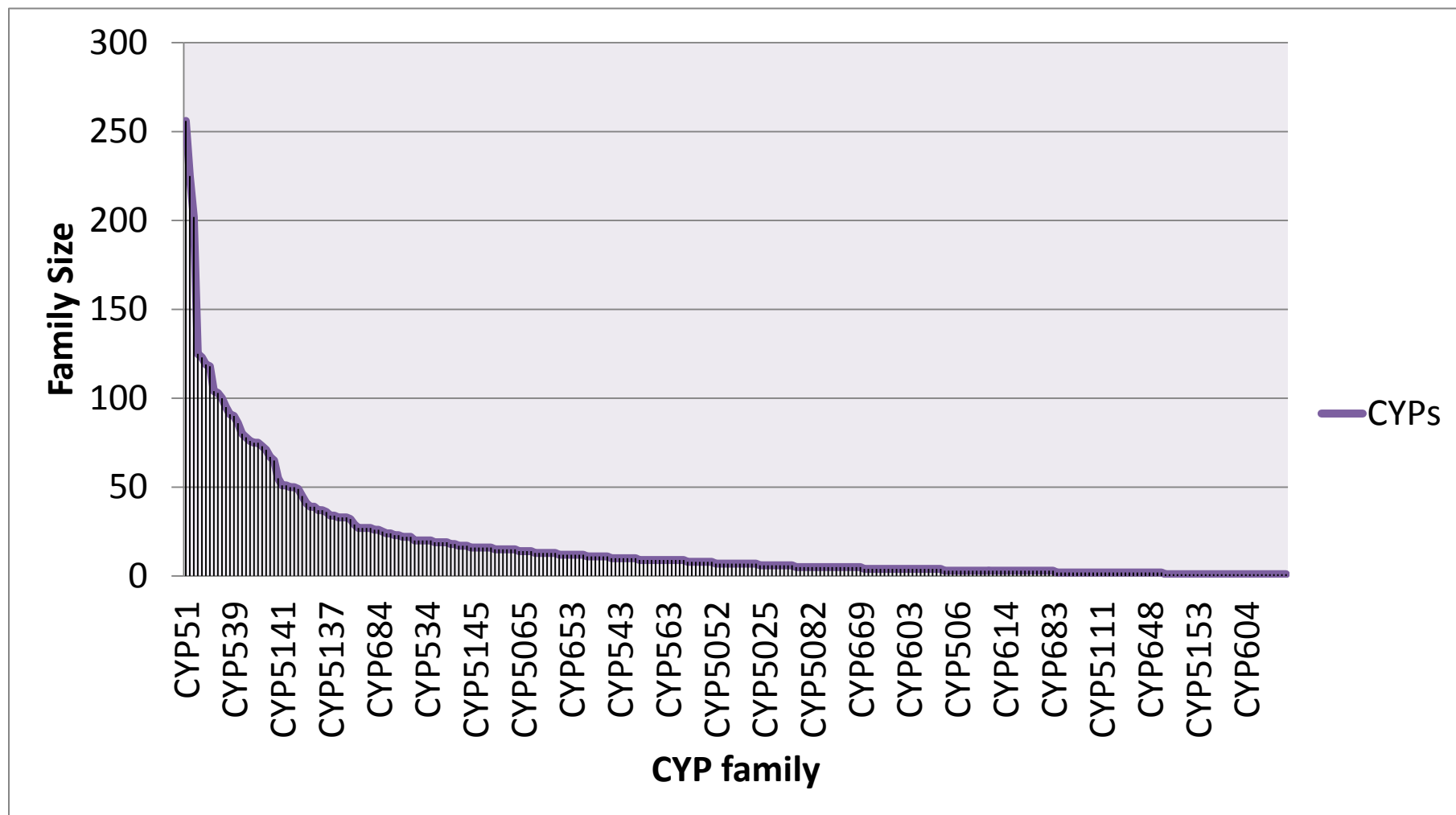

Supplement: Additional file 4 — CYP family sizes follow a power law distribution. The graph shows the family size distribution across families. (PDF 167 kb) [file 1471-2164-13-525-S4.pdf]

## Neighbor joining tree of CYP51 clade

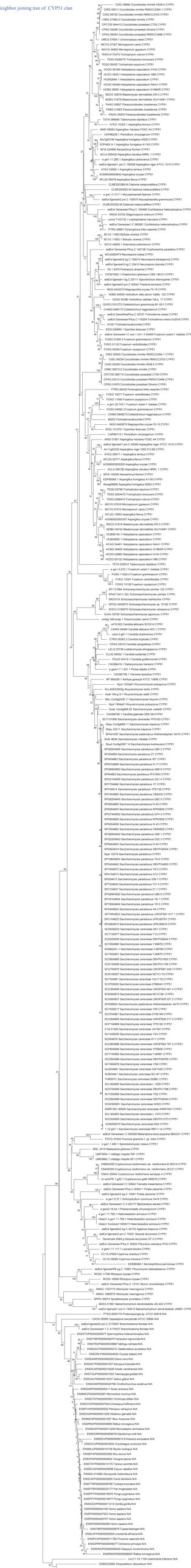

Supplement: Additional file 10 — Neighbor joining tree of CYP51. (PDF 703 kb) [file 1471-2164-13-525-S10.pdf]

Neighbor joining tree of CYP61 Clan

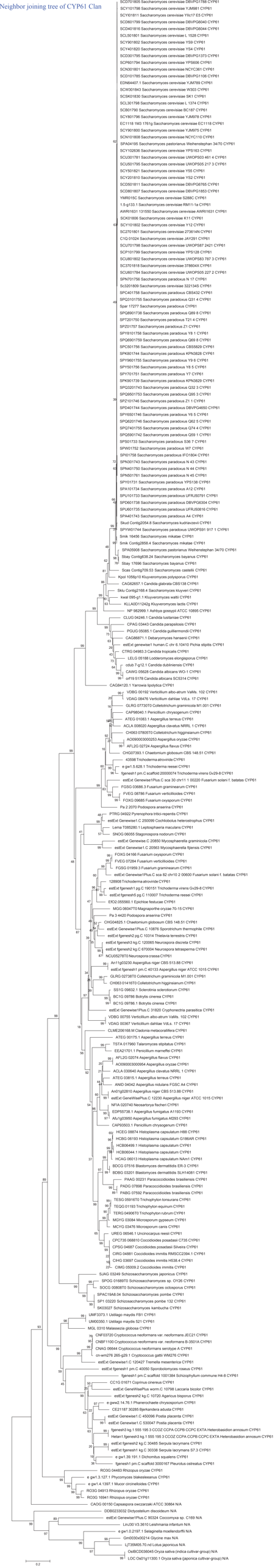

0.2

Supplement: Additional file 11 — Neighbor joining tree of CYP61. (PDF 552 kb) [file 1471-2164-13-525-S11.pdf]

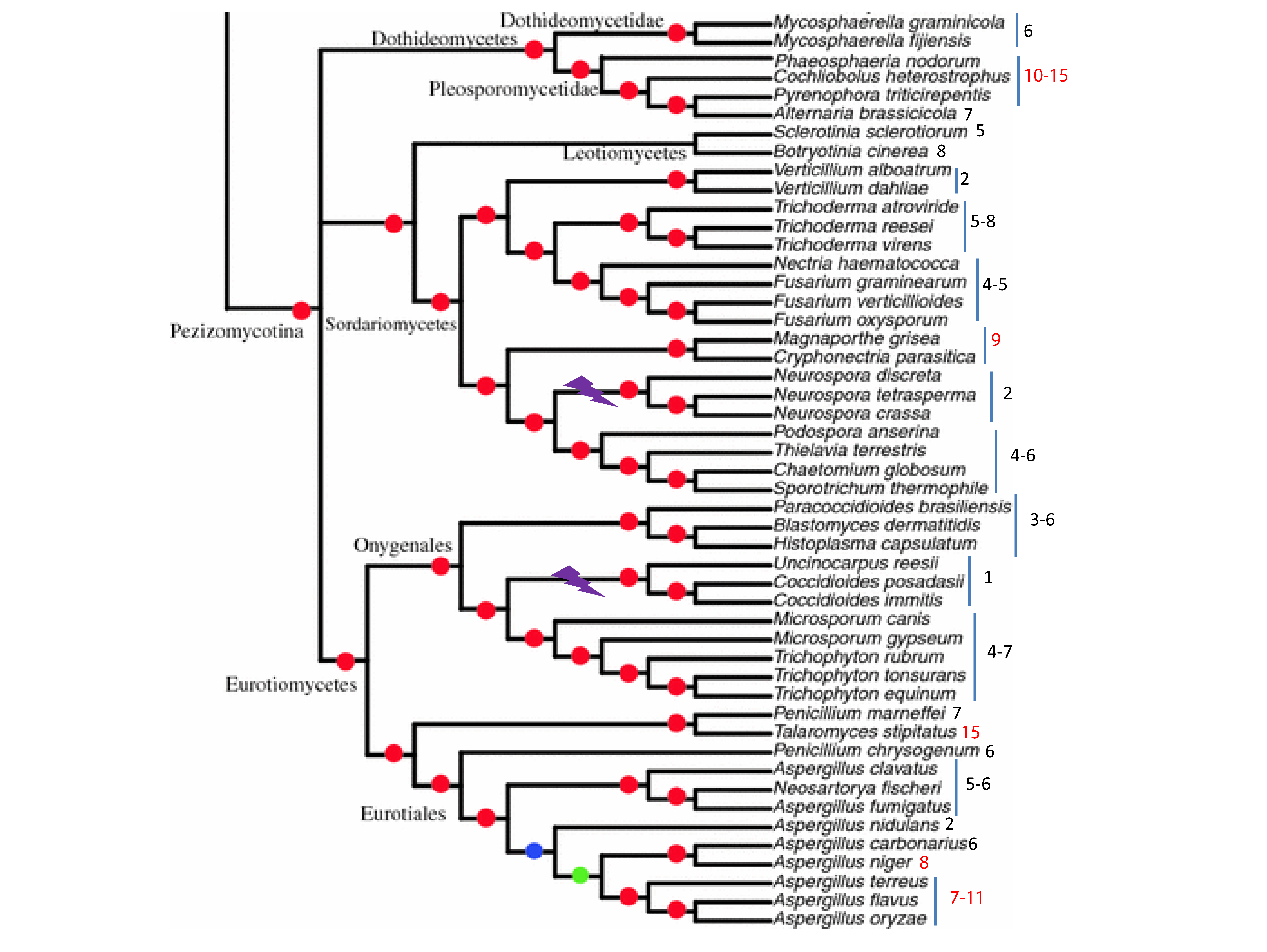

Supplement: Additional file 12 — Phylogenetic tree of CYP65 in Pezizomycotina. Basidiomycetes and Ascomycete yeast species lack family CYP65. In Pezizomycotina, there is large variation in the number of CYP65 family genes. Coccidioides and Neurospora spp. have only one and two members, respectively. On the other hand, Dothideomycetes fungi have 6–15 members. The tree was adapted from Medina et al. [70]. (TIFF 7901 kb) [file 1471-2164-13-525-S12.tiff]

Neighbor joining tree of CYP65 Clan

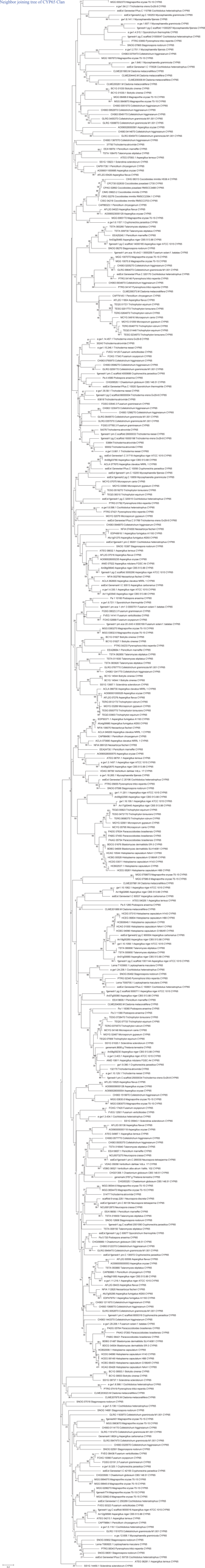

Supplement: Additional file 13 — Neighbor joining tree of CYP65. (PDF 822 kb) [file 1471-2164-13-525-S13.pdf]

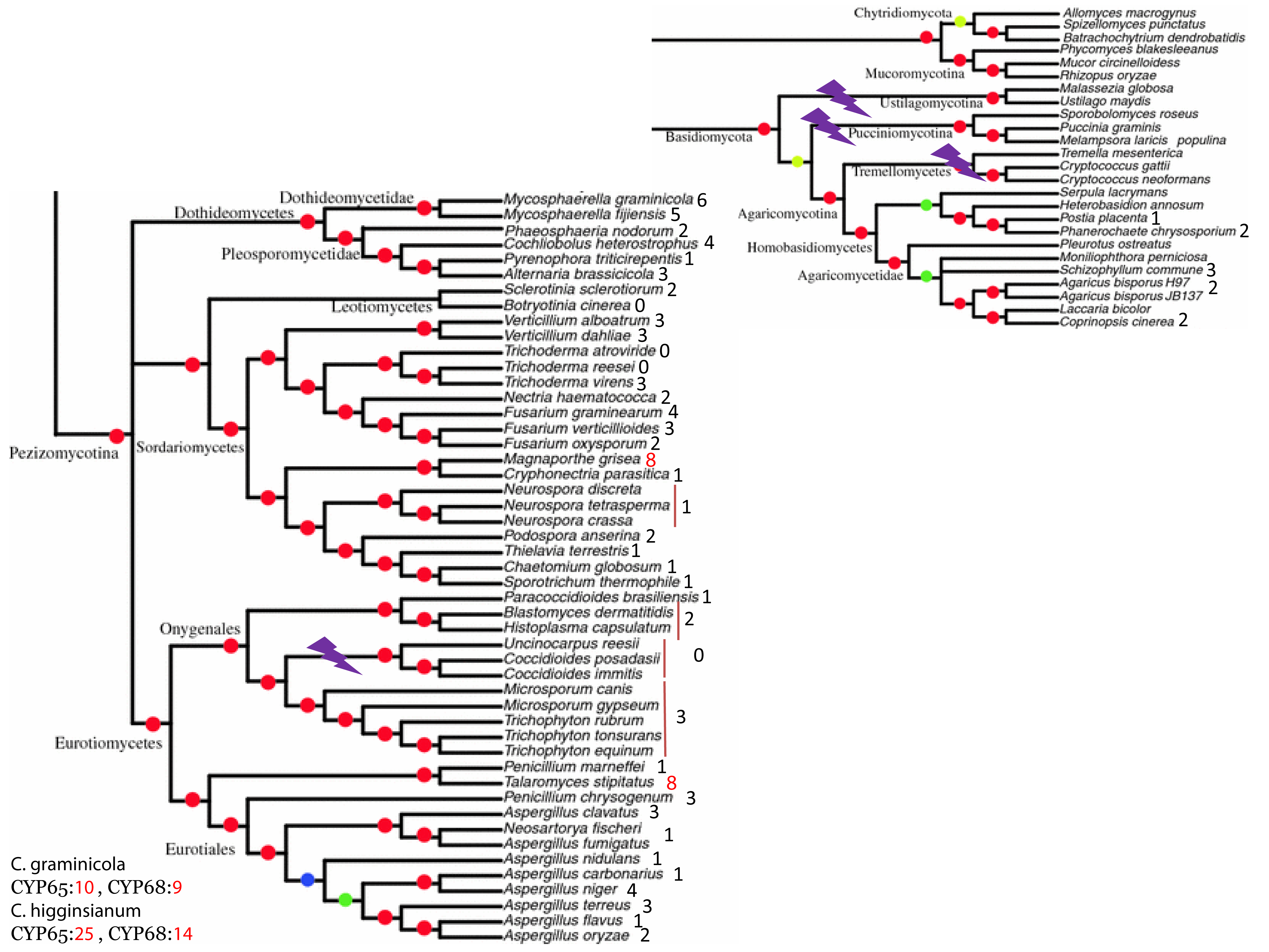

Supplement: Additional file 14 — Phylogenetic tree of CYP68. CYP68 family members are found in a number of secondary metabolism gene clusters. This family was lost in yeasts, and is absent in most Basidiomycetes except for some Homobasidiomycetes species. The tree was adapted from Medina et al. [70]. (TIFF 8118 kb) [file 1471-2164-13-525-S14.tiff]

Neighbor joining tree of CYP505 Clan

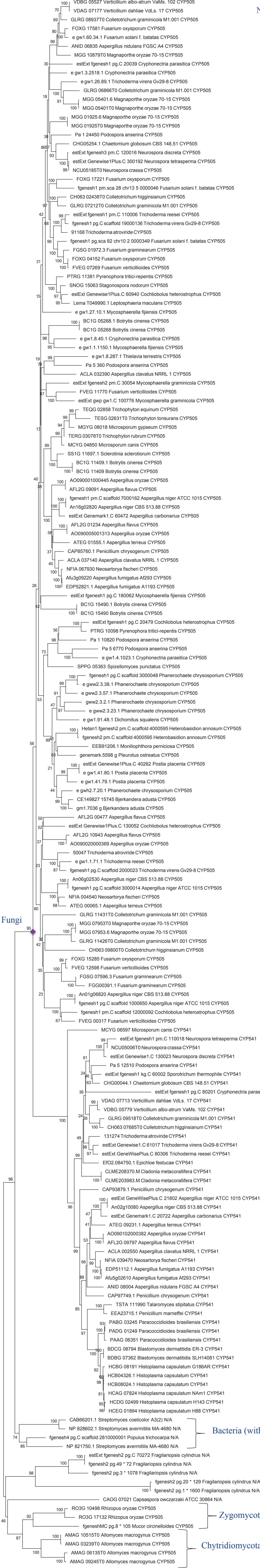

Supplement: Additional file 15 — Neighbor joining tree of CYP505-CYP541. (PDF 507 kb) [file 1471-2164-13-525-S15.pdf]

Maximum likelihood tree of CYP52 Clan

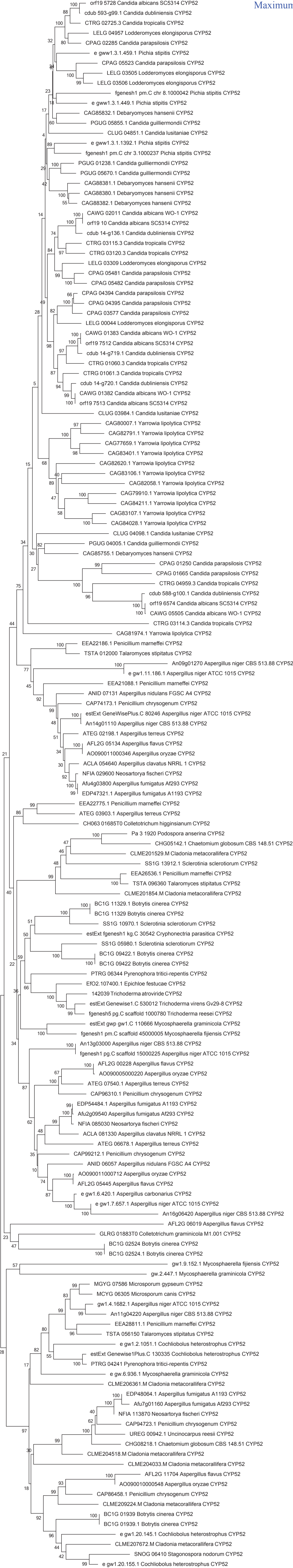

Supplement: Additional file 16 — Maximum-likelihood tree of CYP52. (PDF 431 kb) [file 1471-2164-13-525-S16.pdf]

## Maximum likelihood tree of CYP504 Clade

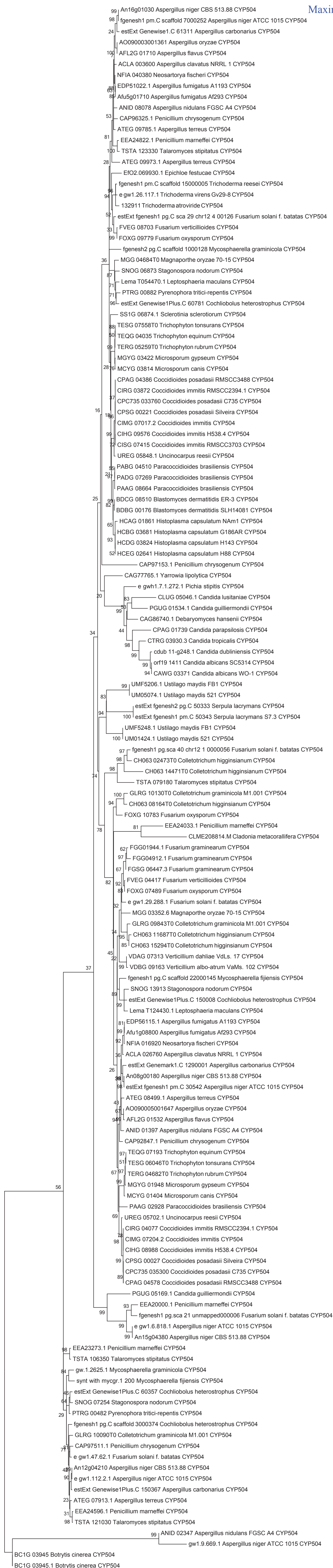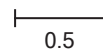

Supplement: Additional file 17 — Maximum-likelihood tree of CYP504. (PDF 395 kb) [file 1471-2164-13-525-S17.pdf]

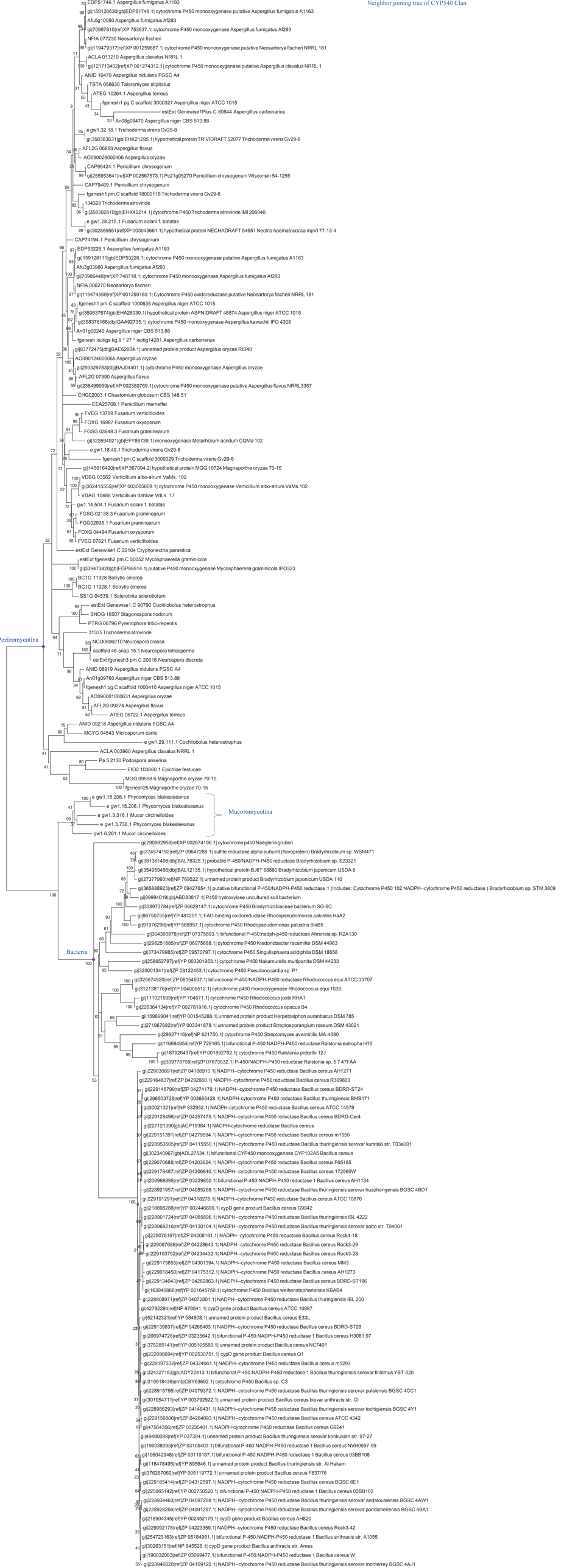

Supplement: Additional file 18 — Phylogenetic tree of CYP540. (PDF 548 kb) [file 1471-2164-13-525-S18.pdf]

Neighbor joining tree of Clan CYP544

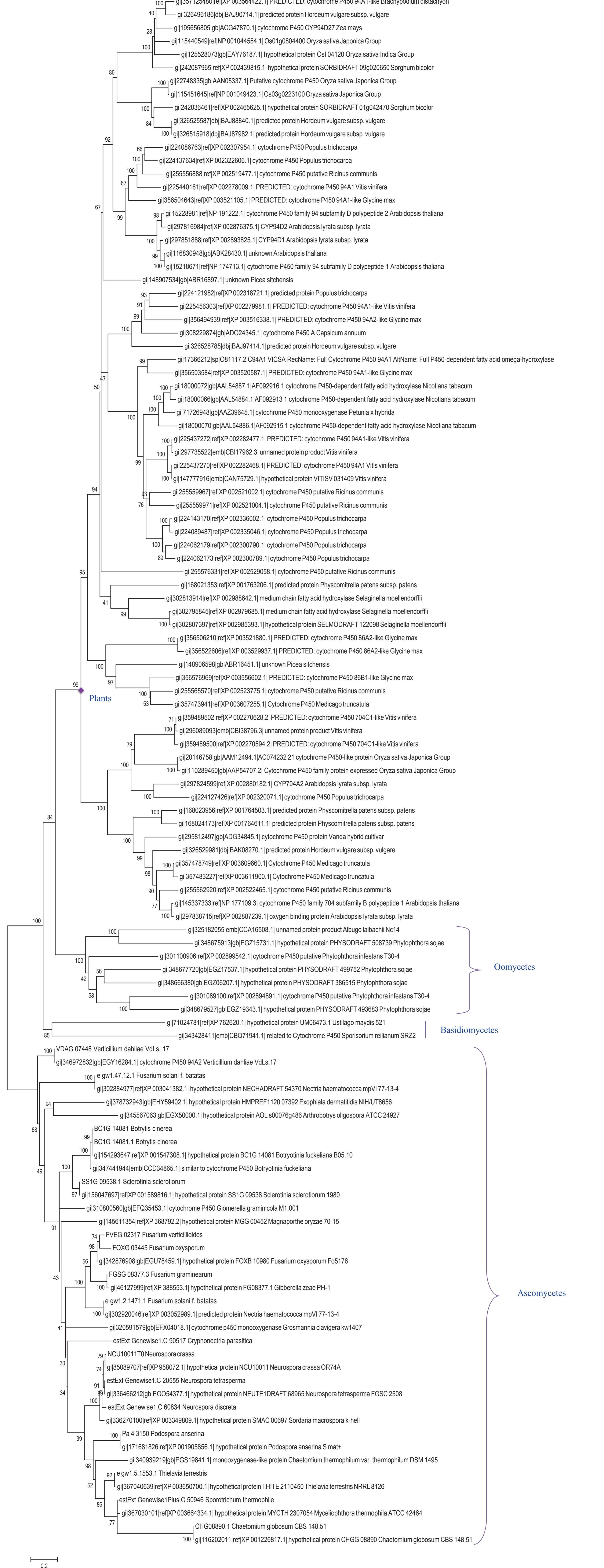

Supplement: Additional file 19 — Phylogenetic tree of CYP544. (PDF 443 kb) [file 1471-2164-13-525-S19.pdf]

Neighbor joining tree of Clan CYP5025

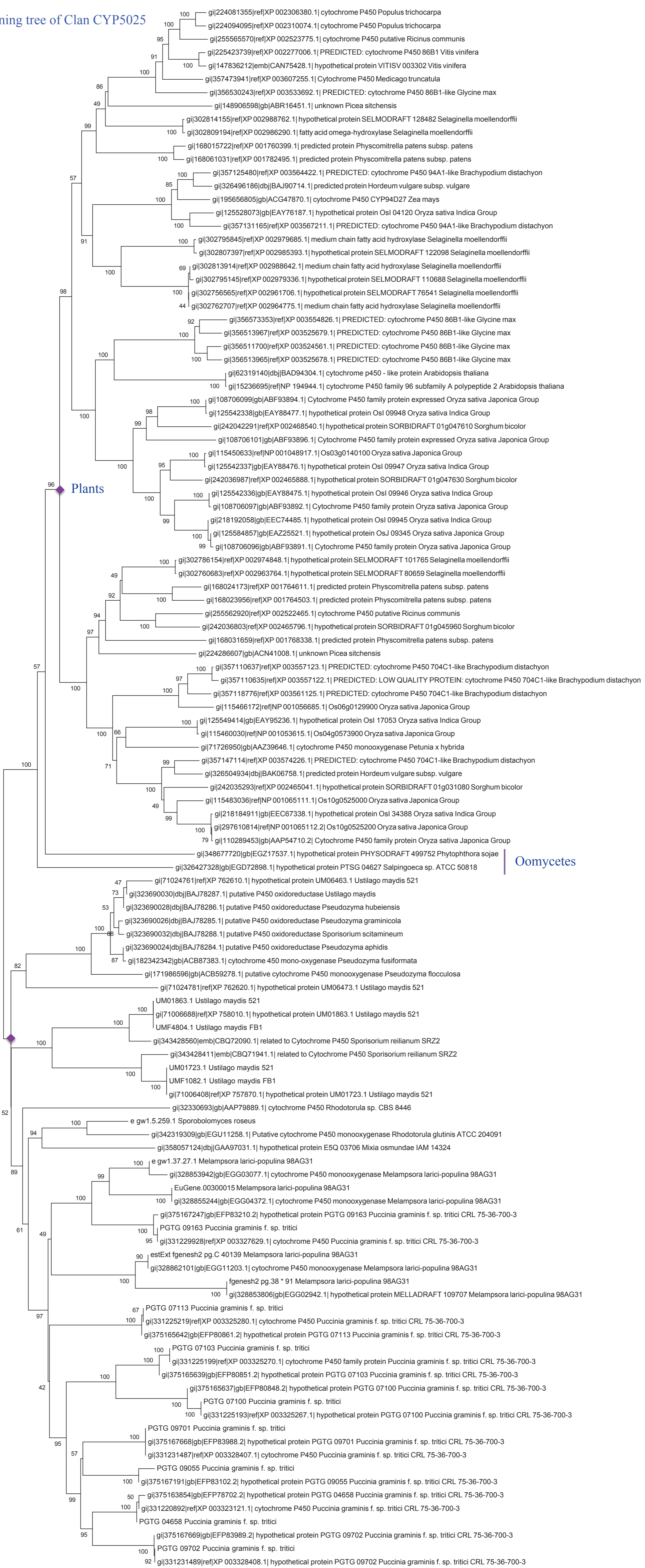

Supplement: Additional file 20 — Phylogenetic of CYP5025. (PDF 437 kb) [file 1471-2164-13-525-S20.pdf]

Neighbor joining tree of Clan CYP645

Bacteria

Fungi

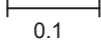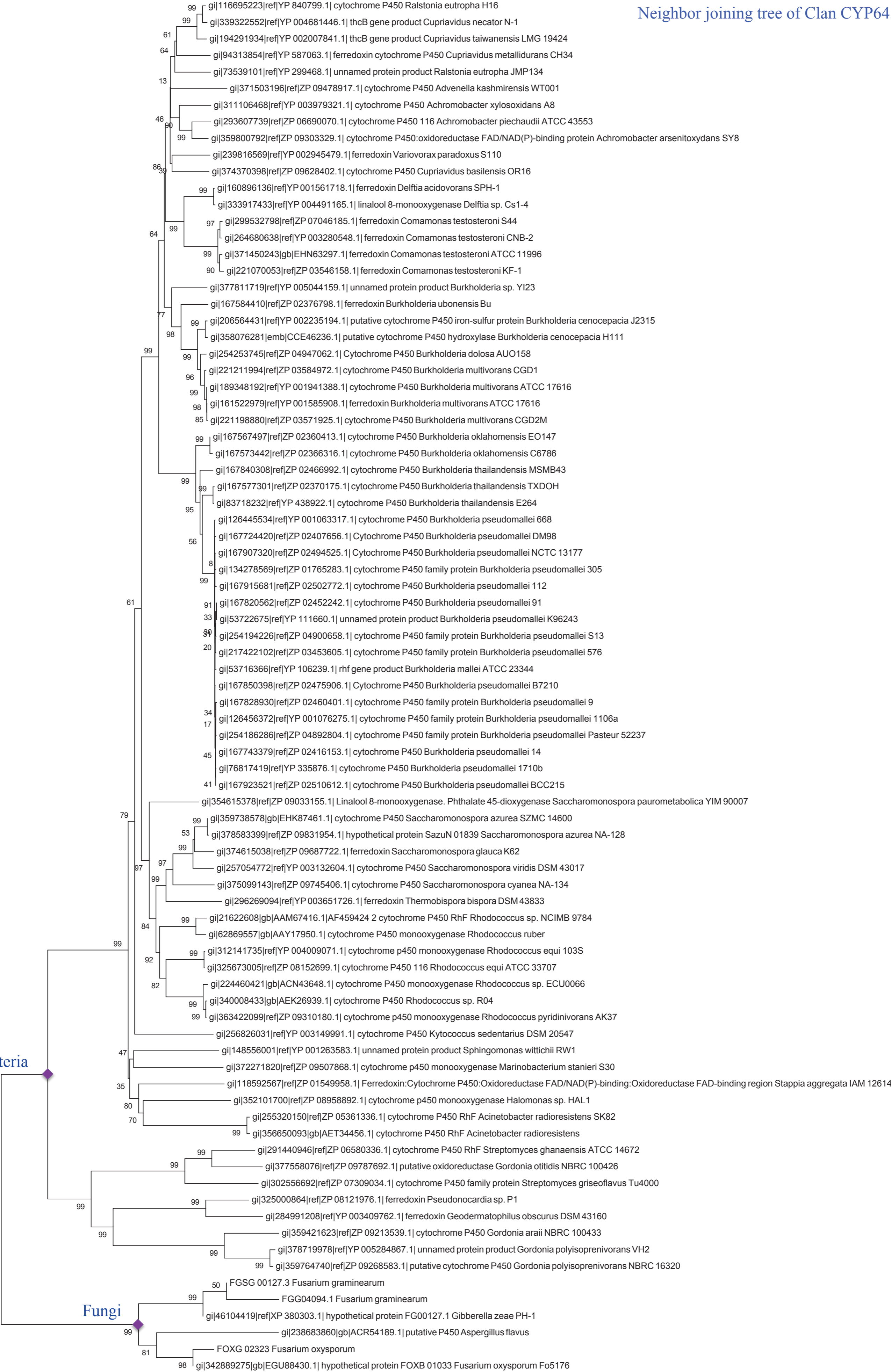

Supplement: Additional file 21 — Phylogenetic of CYP645. (PDF 354 kb) [file 1471-2164-13-525-S21.pdf]
